# Improvement of the Prediction Power of the CoMFA and CoMSIA Models on Histamine H3 Antagonists by Different Variable Selection Methods

**DOI:** 10.3797/scipharm.1204-19

**Published:** 2012-05-24

**Authors:** Jahan B. Ghasemi, Hossein Tavakoli

**Affiliations:** Department of chemistry, faculty of sciences, K. N. Toosi University of Technology, Tehran, Iran.

**Keywords:** Histamine H3 antagonists, Enhanced replacement method, Genetic algorithm, Stepwise multiple linear regression, Successive projection algorithm

## Abstract

The aim of this study is to enhance the predictivity power of CoMFA and CoMSIA models by means of different variable selection algorithms. The genetic algorithm (GA), successive projection algorithm (SPA), stepwise multiple linear regression (SW-MLR), and the enhanced replacement method (ERM) were used and tested as variable selection algorithms. Then, the selected variables were used to generate a simple and predictive model by the multilinear regression algorithm. A set of 74 histamine H_3_ antagonists were split into 40 compounds as a training set, and 17 compounds as a test set, by the Kennard-Stone algorithm. Before splitting the data, 17 compounds were randomly selected from the pool of the whole data set as an evaluation set without any supervision, pretreatment, or visual inspection. Among applied variable selection algorithms, ERM had noticeable improvement on the statistical parameters. The r^2^ values of training, test, and evaluation sets for the ERM-MLR model using CoMFA fields were 0.9560, 0.8630, and 0.8460 and using the CoMSIA fields were 0.9800, 0.8521, and 0.9080, respectively. In this study, the principles of organization for economic cooperation and development (OECD) for regulatory acceptability of QSARs are considered.

## Introduction

One of the most frequently used QSAR techniques is the comparative molecular field analysis (CoMFA) [1–5]. The CoMFA method was developed to take into account the effect of steric and electrostatic interactions, which are involved in blocking a molecule from its receptor. In CoMFA, each molecule is located within grid-spacing through a grid-box dimension, and a probe calculates the energy fields between it and other aligned molecules. In this method, we assume that the whole molecule interacts with the receptor in all directions and the energy fields are then calculated for all of the grids. As a result, thousands of interactions participate in the model. These variables consist of two types: some of them have a correlation with biological activity and the others are noisy variables, which are poorly informative and irrelevant to the biological activities [5]. However, we know from the results of X-ray crystallography of a protein-ligand complex that only some parts of the molecule interact with the receptor [6, 7].

In the literature, there are some solutions to address this problem. First, series are methods that try to improve the quality of CoMFA models by discriminating between informative and meaningless variables. The genetic algorithm and GOLPE are two variable selection algorithms that have been used previously to extract meaningful variables from the large pool of calculated interactions [8, 9]. It is also possible to select a cluster of variables, rather than a single variable, by a smart region definition (SRD) procedure, which is as advanced as the GOLPE algorithm [10]. The prediction-weighted partial least-squares regression algorithm (PWPLS) selects predictor variables and weight them to create a model that is more robust than the CoMFA model [11]. CoMFA region focusing (CoMFA-RF) is another similar attempt to weight the lattice points in a CoMFA region to enhance or attenuate the contribution of these points to the PLS model [12]. In contrast to the first series, there are some methods such as Compass [13], SURFCOMP [14], or CoMSA [15] AFMoC [16] that try to generate variables that are more effective and reduce non-predictive variables. One of the differences between CoMFA and these methods is that they try to sample CoMFA-like fields on the molecular surface or near such a surface. Therefore, the amount of noisy variables decreases. In addition, there are some methods which use receptor information to avoid generation of non-informative variables.

CoMSIA (comparative molecular similarity indices analysis), is developed based on similarity indices. Unlike CoMFA, CoMSIA applies a Gaussian-type distance-dependent function to calculate steric, electrostatic, hydrophobic, and hydrogen bonding donor and acceptor fields [17, 18]. Like CoMFA, CoMSIA uses an atomic probe at regularly spaced grid points around the aligned molecules. Then, the probe experiences a large number of noisy and parametric interactions. On the other hand, it has been proven that variable selection and outlier detection are related. Then the molecules that are chosen as outliers by a set of descriptors may be within the model when described by a different set of descriptors, and also the regression model will be distorted toward the outliers. In addition, as the number of descriptors increases, the risk of chance correlation may increase 19, 20]. An intelligence variable selection with true judgment between informative and noisy variables could generate an ideal model, which is predictive, robust, and has no molecule labeled as an outlier with it. In this study, GA, SPA, SW-MLR, and ERM were applied on the CoMFA and CoMSIA fields. Then the selected variables were modeled by the MLR algorithm to generate a simple and predictive model. The performance of the different CoMFA and CoMSIA models were evaluated by modeling a data set of histamine H_3_ antagonists.

Histamine is a biogenic amine neurotransmitter, which interacts with four types of G protein-coupled receptors (GPCR)s i.e. H_1_, H_2_, H_3_, and H_4_ [21]. The GPCRs contain three common parts: seven α-helices that span the cell membrane, an extracellular N-terminus part and a cytoplasmic C-terminus part with variable length. The third and fifth transmembrane (TM) regions of receptors are involved in ligand-drug interactions, while the third intracellular loop is responsible for a signaling pathway connection [22, 23]. The Histamine H_3_ receptor (HH3R) was initially identified on a pharmacological basis by Arrang et al in 1983 [24]. In 1999, Lovenberg et al cloned this receptor (GPCR97, Uniport ID: Q9Y5N1). GPCR97 has (31%) homology with the a_2_-adrenergic and muscarinic M_1_receptors, whereas 22% and 21.4% are homologous with the H_1_ and H_2_ receptors, respectively. The sequence of GPCR97 has a 445-amino acid coding region with a notable aspartic acid residue in transmembrane region 3, which is a putative binding site for the interaction of receptors with primary amines [25].

The new generations of HH3R antagonists are non-imidazole based. They contain at least one basic amine, either a piperidine or pyrolidine, which is connected by an alkyl linkage to an aromatic ring. However, antagonists with a second basic site show significantly better activity, such as the ligands in this study [26]. The interaction of the negatively charged carboxylic group of Asp114 on the third helix of the HHR3 and a protonated amine group of an antagonist, is the common point in all of the docking results of HHR3 antagonists by different homology modelling [27–31].

HHR3 antagonists act on both the histaminergic and non-histaminergic neurons. On the histaminergic neurons, they regulate the release of histamine and its synthesis and on the non-histaminergic neurons, they presynaptically inhibit the release of a number of other neurotransmitters such as dopamine [32], GABA [33], acetylcholine [34], noradrenaline [35], and serotonin [36]. The H_3_ receptor antagonists are involved in cognition, sensory gating, food intake, sleep, the waking state, and pain perception. Thus, this could be a potential target for the treatment of numerous diseases, disorders affecting cognition (e.g., attention deficit and hyperactivity disorder [ADHD], Alzheimer’s disease, and schizophrenia), sleep (e.g., hypersomnia and narcolepsy), and energy homeostasis (e.g., obesity), myocardial ischaemia, migraine, and inflammatory diseases [37–40].

## Results and Discussion

### Comparison and validation of the models (Goodness-of-fit, robustness, predictivity)

**Fig. 1:**
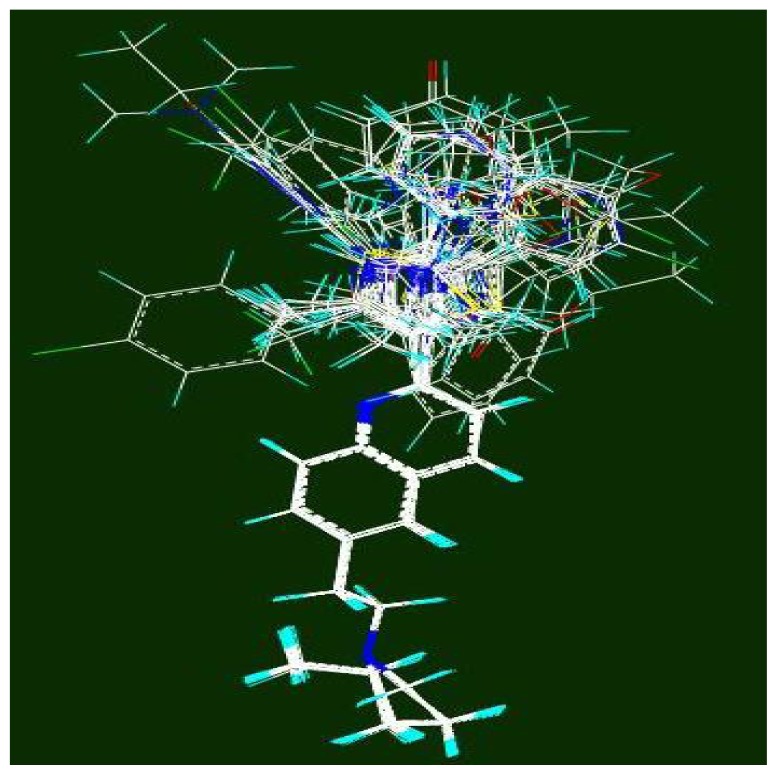
The aligned compounds based on the most active compound (51) in the orientation achieved by AOS

The CoMFA model which was built by PLS in SYBYL showed very poor statistical parameters (e.g. q^2^~0.1). In addition, its results were sensitive to the orientation and placement of the compounds in the box. Therefore, the all-orientation search (AOS) and the all-placement search (APS) strategies [41] applied on the aligned compounds to improve the q^2^ value. Using the AOS algorithm, all of the possible samplings of the molecular field are tested by systematically rotating and translating the molecular aggregate within the grid, and subsequently the one with the highest q^2^ value can be picked out. The AOS algorithm was run in 30, 10, 5, 1, and even 0.1º intervals (Fig. 1), in such a way that the result of each AOS run was fed to the next run. In APS, aligned molecules moved in the box in all three dimensions of space and the best placement was selected according to the highest q^2^. The best APS results did not represent significant changes in the q^2^ value by 1.00, 0.50, 0.10, and 0.05 Å movements of the aligned compounds.

One of the most important aspects of a QSAR model is its predictivity. It is so important that the OECD member countries adopted it as a separate and critical principle for an ideal model [42]. Tropsha et al have emphasized that having such a high value for goodness-of-fit and cross-validated correlation coefficient r^2^ (q^2^) is insufficient for judging about the predictivity power of a model. Although a high q^2^ value is vital, it cannot guarantee the predictivity power of a model [43, 44]. Therefore, an external test set is necessary. An ideal QSAR model must also have accurate predictivity on the external set [45]. Therefore, we selected 17 of 74 compounds in a fully blind sampling for the independent or evaluation set and the remaining 57 compounds were divided into 40 compounds as the training set, and 17 compounds as the test set by the Kennard-Stone algorithm [46]. The Kennard-Stone algorithm tries to guarantee uniform selection of objects for the training and test sets. The r^2^ and q^2^ values of the CoMFA model on the AOS-aligned compounds were 0.9780 and 0.6040, respectively (Table 1). The CoMFA-RF algorithm improved the q^2^ value to 0.6530 by weighting CoMFA fields. Although it improved the statistical parameters of the CoMFA model to some extent, satisfactory results were still not obtained. Hence, the raw fields were extracted from SYBYL. The zero columns were removed. Then different variable selection algorithms were applied to the rest of 3331 CoMFA fields to filter out the noisy variables. Variable selectors have more of a tendency to sterically clash with CoMFA fields than electrostatic ones, because of their variance contribution. Then CoMFA standard scaling was applied to the CoMFA fields to avoid swamping the electrostatic fields with steric ones. This is a block-scaling and in the case of CoMFA and CoMSIA fields, this is the best one.

The PLS algorithm performs regression on the latent variables which do not have physical meanings, but the MLR algorithm is simpler and more interpretative than the PLS algorithm. However, due to the collinearity between the CoMFA or CoMSIA fields, MLR disables to generate a successful model especially from a huge amount of variables. Then using a variable selector to extract informative variables with multiple linear regression for building a simple and easy to interpret model, will be useful. Among the variable selectors, which were applied on the extracted CoMFA fields, the results of SW-MLR were significantly better than SPA and GA, and the results of the SPA algorithm were better than GA to some extent (Table 1). In spite of improving the predictivity power of the models by these variable selectors, they could not give acceptable predictivity power according to following measures:

$$\begin{array}{l} {{\text{r}}^{2}}_{\text{CV}}>0.5\\ {{\text{r}}^{2}}_{\text{Pred}}>0.6\\ ({{\text{r}}_{0}}^{2}-{\text{r}}^{2})/{\text{r}}^{2}<0.1\;\text{and }0.85<\text{k}<1.15\;\text{or }({\text{r}}^{2}-{{{\text{r}}^{\prime }}_{\text{0}}}^{2})/{\text{r}}^{2}<0.1\;\text{and }0.85<{\text{k}}^{\prime }<1.15\\ |{{\text{r}}_{0}}^{2}-{{{\text{r}}^{\prime }}_{\text{0}}}^{2}|\;<0.3 \end{array}$$

The r_o_^2^ and r′_o_^2^ are the correlation coefficients of predicted versus observed activities for regressions through the origin and vice versa. The k and k′ values are their corresponding slopes, respectively [43].

**Fig. 2:**
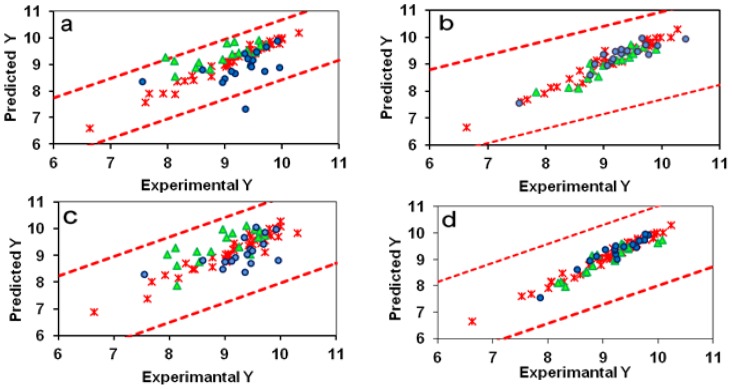
Predicted versus experimental bioactivities for (a) CoMFA model, (b) ERM-MLR model based on the CoMFA fields, (c) CoMSIA model and (d) ERM-MLR model based on the CoMSIA fields; The molecules in the training, test set, and Evaluation sets are presented in stars, triangles, and circles respectively. The dotted lines indicate the ±2S margins

**Fig. 3:**
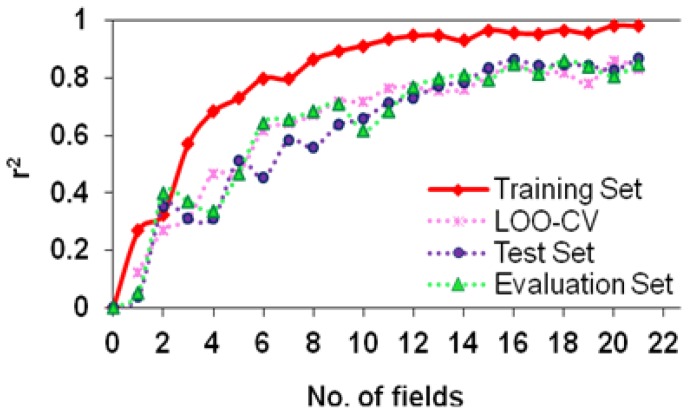
Using 16 CoMFA fields result in simultaneously maximization on the ERM-MLR model features (the r^2^ values of the training, LOO-CV, test, and evaluation sets)

The SW-MLR model does not meet all the above measures because the k′ value for this model is smaller than 0.85. However, the statistical parameters for this model, especially its r^2^ value for the evaluation set, are acceptable. This model with 15 variables had an r^2^ value of 0.9059, a q^2^ value greater than 0.5 (0.6071), and a fair r^2^ value of 0.7527 for the test set. Therefore, we considered this model as a predictivity model (Table 1). The ERM algorithm donates such a priority to the subsequent MLR model, which distinguishes it from the other models. The goodness-of-fit value (0.9560) for this model with 16 variables is as high as this value for traditional CoMFA or CoMFA-RF models, which have the advantage of the PLS algorithm. In addition, the high q^2^ values of leave-one-out (LOO) and leave-many-out (LMO) cross validation (10 groups) for this model (i.e. 0.8810 and 0.8700, respectively) emphasize that this model is very close to an ideal predictive 3D QSAR model. The considerable improvement of about 0.4 and 0.5 units, respectively, in the r^2^ values of the test and evaluation sets over the traditional CoMFA model were obtained for the ERM-MLR model. In addition, ERM-MLR passes all of the predictivity measures successfully (Table 1). Figure 2(a) and (b) show predicted versus experimental biological activities for the traditional CoMFA and ERM-MLR model based on the CoMFA fields. For the traditional CoMFA model, some of the predicted y values show a clear bias from the experimental ones, and two objects detect as outliers because their predictions are located beyond the ±2S boundary lines. However, in Fig. 2b all predicted y values are located within ±2S boundary lines. Then in the ERM-MLR model, no molecule is labeled as an outlier. These results, besides the low RMSEP value (0.2258) for the ERM-MLR model, show that among all variable selector algorithms, ERM is the most effective algorithm and acts as a semi-full search tool. Figure 3 shows that with 16 variables, the built MLR model, besides simplicity, has remarkable statistical parameters and the r^2^ values for the training, test, and evaluation sets are the highest. The generated ERM-MLR model is a combination of the selected CoMFA fields:

$$\begin{array}{l} \text{Biological activity}=0.2993{\text{S}}_{876}+0.4488{\text{S}}_{521}+0.9246{\text{S}}_{142}-0.3553{\text{S}}_{1067}-0.2996{\text{E}}_{1087}+\\ 0.3724{\text{E}}_{2727}+1.3755{\text{S}}_{986}-0.5288{\text{S}}_{1087}-0.9670{\text{E}}_{2836}+0.3130{\text{S}}_{842}-0.5613{\text{S}}_{670}+\\ 4.2289{\text{E}}_{2389}-3.5361{\text{S}}_{1221}-0.3647{\text{S}}_{1130}-1.0753{\text{E}}_{2849}-1.2007{\text{S}}_{795} \end{array}$$

**Fig. 4:**
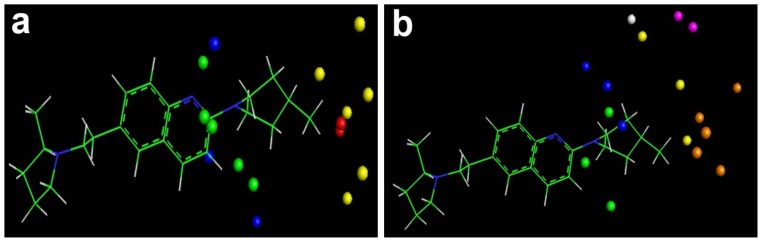
The selected fields by ERM algorithm. a) The selected CoMFA steric (favored green points and unfavored yellow points) and electrostatic fields (favored blue points and unfavored red point); b) The selected CoMSIA steric (favored green points and unfavored yellow points), electrostatic (favored blue points), hydrogen-bond acceptor (favored magenta points and unfavored orange points) and hydrogen-bond donor (unfavored white point)

**Fig. 5:**
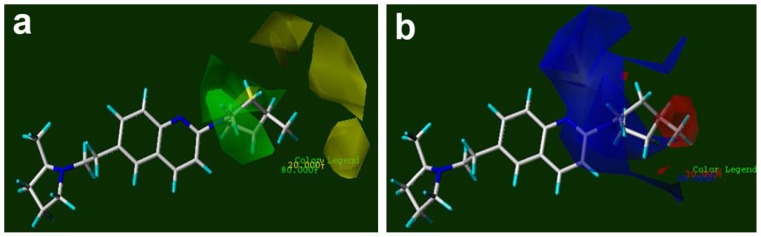
Contour maps of CoMFA-RF based on compound 51: (a) steric, (b) electrostatic fields. (Contours for traditional CoMSIA model not shown here)

Thirteen of the sixteen selected fields are steric and the rest of them are electrostatic, i.e. the contribution of steric fields is more than that of electrostatic ones (Figure 4a). In the MLR algorithm, coefficients of the fields and their signs appear in the equation. As a result, their results are easier to interpret than those of the PLS algorithm. However, the nature of the CoMFA fields is energy and they are calculated by the summation of steric and electrostatic interactions over the whole of the compound. Hence, calculation of energy in different grids may result in identical or similar values. In addition, information from atoms and molecular features are convoluted in fields. Therefore, in practice the interpretation and suggestion of functional groups for various positions on a given scaffold or reconstructed molecule from fields is difficult. Figure 4a illustrated the ERM-selected steric and electrostatic CoMFA fields. These points are to a great extent in agreement with CoMFA (not shown here for simplicity) or CoMFA-RF contour maps (Fig. 5). By this similarity, we can say that the interpretation of these fields is very similar and/or the same with what we can say for that of CoMFA-RF.

Figure 5 shows the contours of CoMFA-RF for the steric and electrostatic maps. Greater values of bioactivity correlate with more bulk near green; less bulk near yellow; more positive charge near blue and more negative charge near red. The contour map of the steric fields has two separate parts: a green part near the backbone and a yellow part far from it. By replacing each compound with another in the space of contours, we can see that the chain substitute, or five and six-membered monocycle substitutes, usually oriented toward the green contours and most of the fused or bridged bicycle substitutes directed toward the yellow areas. The green contour near the backbone indicates that more bulky groups are favorable. It explains why the activity of compound 22 (pIC_50_=9.25) with two methyl groups is higher than that of 23 (pIC_50_= 8.74) with a bromide branch. The same reason is acceptable for higher activity of 21 (pIC_50_=9.72) compared to 22 (pIC_50_=9.25) or 33 (pIC_50_=8.60) rather than 32 (pIC_50_=8.29), which in these pairs a smaller oxygen atom was replaced by a more bulky sulfur atom. The bi-cyclic fused substitute in 78 (pIC_50_=8.44) is located near the green contour, therefore replacing it with a bulky three-cycle substitute in 79 (pIC_50_=9.00) which has increased the activity. The COOEt group in compound 24 increases the activity (pIC_50_=8.15) but this group decreases the activity in compound 25 (pIC_50_=7.46); this shows that the attachment position of a substitute to the backbone is also important, because it results in a different direction of a substitute toward the yellow or green contours. In compound 24, the bulky COOEt substitute oriented toward the green contours, but in 25 oriented toward the yellow contours. The bulkiness of substitutes along the yellow contours causes unfavorable effects on the pIC_50_ values. This is due to the fact that the activity in compound 36 is less than 35 (or 43 < 44, 64 < 63, 69 < 67 < 68). More examples can be found in the data set. It must be noted that, since Figure 5 illustrates the contour maps that were achieved from all of the compounds, then some cases can be found that have incomplete adaptation to the contour maps.

The electrostatic contours of compounds also have two parts. The first part consists of blue contours that are enclosed or are near to the quinoline ring and the second part consists of red contour maps far from the backbone (Fig. 5b). The electronegative groups that oriented toward the red region increase the activity. Therefore compound 14, which has a CN group near the red contour, has higher activity (pIC_50_=9.16) than compound 15 (pIC_50_=8.13), in the same way 35 > 34 and 43 > 42. Again, the attachment-position of a substitute to a compound is important because different attachment-positions change the orientation of the attached group toward the red or blue contours. In such a way, compound 31 has a higher pIC_50_ (9.08) than compound 32 (8.29), because two nitrogen atoms in compound 31 oriented toward the red region, but the oxygen atom in compound 32 oriented toward the blue contour, which by even rotating around the sp^3^ band, its orientation does not differ. Compound 45 has the lowest pIC_50_ value in the set, because its three electronegative fluorine atoms and the oxygen atom of the OH branch directed toward the blue contours. In general, bulky and electropositive groups near the backbone, and small and electronegative groups far from the backbone are favorable in increasing bioactivity.

**Fig. 6:**
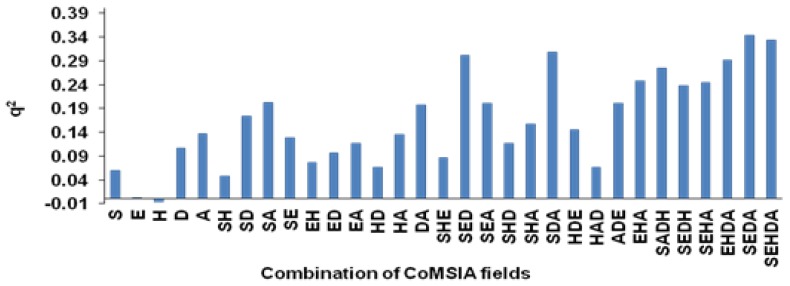
The distribution of q^2^ values that were obtained from 31 different combinations of CoMSIA fields

Table 2 contains the statistical parameters for the traditional CoMSIA and the other models, which benefit variable selections before using MLR. CoMSIA analysis was performed by six components at a column filtering of 1 kcal/mol and grid spacing of 2 Å. To select the optimal CoMSIA results, different combinations of CoMSIA fields were tested (Fig. 6). The combination of steric (S), electrostatic (E), hydrogen bond donor (D), and acceptor (A) fields generated the highest q^2^ (0.3440) and a non-cross-validated r^2^ of 0.9360. Because of these poor statistical results, the CoMSIA fields (SEDA) were extracted from SYBYL. Then zero variables were removed. Block (CoMFA) scaling applied to the rest of the 3478 variables. Finally, different stochastic and systematic variable selectors were applied to them. The selected variables were used in different MLR models. Among these variable selectors, GA and SPA did not have satisfactory results. The r^2^ values of the training, test, and evaluation sets for the stepwise algorithm were 0.8789, 0.7218, and 0.6884, respectively. These results, besides a q^2^ value greater than 0.5 units (0.5900), show that the stepwise algorithm is effective on the quality of the MLR model. Although, these results were statistically acceptable; however, the results of the ERM-MLR model were excellent and dramatically better than those of other models. ERM selected six steric, three electrostatic, one hydrogen bond donor, and seven acceptor fields. The combination of these fields in MLR algorithm results in:

$$\begin{array}{l} \text{Biological activity}=-0.0523\;{\text{A}}_{3366}-2.5960\;{\text{A}}_{2541}+0.4903{\text{S}}_{762}+0.2354{\text{A}}_{2978}-0.2096{\text{A}}_{3089}\\ -0.2765{\text{D}}_{2232}-1.5016{\text{S}}_{428}-0.4413{\text{A}}_{2952}-0.4516{\text{S}}_{523}+4.4938{\text{S}}_{104}+0.8839{\text{E}}_{1649}-\\ 2.0127{\text{A}}_{2822}+0.2209{\text{E}}_{2067}+0.2052{\text{E}}_{1997}+3.1509{\text{S}}_{555}+0.1573{\text{A}}_{3203}-0.5170{\text{S}}_{914} \end{array}$$

**Fig. 7:**
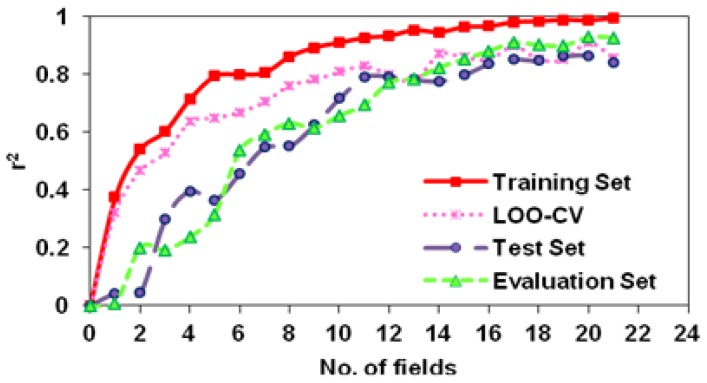
Using 17 CoMSIA fields result in simultaneously maximization on the ERM-MLR model features (the r^2^ values of the training, LOO-CV, test, and evaluation sets)

The r^2^ values of the training, test, and evaluation sets and the q^2^ value of LOO-CV for this model were 0.9800, 0.8521, 0.9080, and 0.8970, respectively. Then the ERM-MLR model has a 0.3-unit increase in the q^2^ value over the traditional CoMSIA model. Here an effective variable selector improved a non-predictive model (traditional CoMSIA) to a predictive one (Table 2). In addition, ERM is a powerful variable selector by participating with the informative variables in the model, which are highly correlated by y, causing all of the molecules to fall in the model space. Hence, the ERM-MLR model does not label any molecule as an outlier, and decreases dispersion in the predicted values (Fig. 2(c) in comparison with 2(d)). Figure 4(b) is a visualization of the selected CoMSIA variables. Greater values of bioactivity are correlated with more bulk near green points and less bulk near yellow points. Magenta colored points indicate points where hydrogen-bond acceptor groups increase activity; orange points represent the orientation that inserting hydrogen-bond acceptor groups decreases activity. The blue and red points show the locations where electropositive and electronegative groups are favored and unfavored, respectively. The greater contribution of the orange-magenta points (0.41% of total selected fields that selected by ERM) and the green-yellow points (35%) compared to the blue points, show that steric and hydrogen bond acceptor fields are more important in the model than electrostatic fields (18%). Since using 17 CoMSIA fields results in simultaneously increasing the r^2^ values of the training set, LOO-CV, test, and evaluation sets, then these number of fields were regarded as the optimum number of variables, which must participate in model building (Fig. 7).

### The Applicability Domain (AD)

**Fig. 8:**
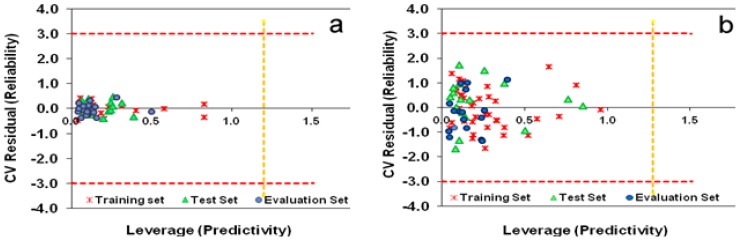
The domain of Applicability of ERM-MLR on the CoMFA fields (a) and the CoMSIA fields (b). The vertical lines indicate warning leverage

The domain of applicability is a space that is generated by the descriptors of the training set and corresponding biological values. If the predicted biological activity for a compound falls within this domain, it is not extrapolated by the model and then is reliable [47]. A William plot is a useful tool for the simultaneous investigation of AD and outlier detection. It is a visualization of predictivity (standardized cross-validated residuals) versus reliability (leverages). In this plot, moving from the origin toward the x direction will increase the unreliability of the predicted values, and moving toward the y direction will decrease the predictivity of the model (Fig. 8). These figures show that the selected variables were so successful that no molecule labeled as an outlier in the ERM-MLR models were based on the CoMFA and CoMSIA fields.

### Progressive scrambling analysis (PSA)

Progressive scrambling analysis is a test for investigating the robustness of a QSAR model and its sensitivity to chance correlations. In a large data set, some members may be twins together. Then in leave-one-out cross validation, a near twin of each left-out compound may remain in the training set. Hence, LOO-CV is not a good criterion for the robustness of a model. In addition, instead of shuffling the responses through the whole rang such as what the y-randomization algorithm does, PSA scrambles responses only within the blocks across the range. Then PSA is sensitive even to small perturbations in the data set [48]. In our study, PSA is run more than 30 times to decrease its dependency on the random number seed. The minimum and maximum of bins were two and 10, respectively, and the critical point was set to 0.85. The q^2^ values of scrambled y for traditional CoMFA, ERM-MLR (based on CoMFA fields), and ERM-MLR (based on CoMSIA fields) models were 0.4056, 0.1683, and 0.1590, respectively, and their calculated cross-validated standard error (cSDEP) values were 0.6452, 0.7691, and 0.7748 for 30 PSA runs, respectively. The low q^2^ values show that models that were constructed after variable selection algorithms do not suffer chance correlation.

## Experimental and Methods

### A defined end point (biological activity)

The first item of the EOCD principles states that for having an ideal QSAR model, a well-defined end point based on a standardized test protocol is necessary [42]. Recently, Liu et al synthesized a series of quinoline compounds via the Friedlander quinoline condensation and assessed their binding affinities by an identical test protocol (displacement of [3H]-N-a-methyl histamine, using cloned human H_3_ receptors). All of the reported values are the average of three independent measurements and the standard errors of the mean were less than 0.25 in each case (Table 3) [49].

### Geometry Optimization, Alignment and CoMFA/CoMSIA fields’ calculations

The IC_50_ values (nM) of the 74 compounds were converted to a logarithmic scale (pIC_50_) before modeling. The CoMFA and CoMSIA fields were calculated by the SYBYL 7.3 molecular modeling package (Tripos, Inc, St. Louis, USA) running on a Red Hat Linux workstation 4.7. The most active compound (i.e. compound 51) was selected as a template and other compounds were superimposed according to their common structure. The accuracy of the prediction of CoMFA and CoMSIA models and the reliability of the contour maps depend on the structural alignment of the molecules. Rigid-body aligned molecules were performed using maximum common substructures defined by the Distill method (with included bond types in rings). Distill alignment had suitable results on this dataset. The aligned set of the molecules were positioned inside a 3D cubic lattice of a 2 Å (default distance) spacing grid box with an extension of 4 Å units in all Cartesian directions beyond the molecules to envelop all of them. The interaction energies for each molecule were calculated at each grid point using different probes i.e. C (SP^3^), O, N, etc. probes. The best results were achieved by a sp^3^ hybridized carbon atom with a +1 charge. The partial atomic charges were calculated by the Gasteiger–Hückel method and energy minimizations were performed using the Tripos force field with a distance-dependent dielectric and the Powell conjugate gradient algorithm (convergence criterion of 0.01 kcal/mol Å) in order to obtain the best conformer for each molecule. Interaction of the probe with the molecules on a 2 Å grid provided 1800 explanatory variables for each field per compound. The uninformative values were removed by an optimized column filtering value equal to 1.8 kcal/mol for CoMFA and 1.0 kcal/mol for CoMSIA models. For applying variable selection and MLR algorithms, fields’ entries were extracted from SYBYL by two separate SPL scripts for CoMFA and CoMSIA fields. All other parameters were set as defaults.

### Variable selection strategies

#### Genetic algorithm

The genetic algorithm was inspired by a natural process. It tries to select the best-fitted variable with the higher fitness function through exploitation (natural selection) and exploration (evaluation) process. It benefits genetic operators (mutation and recombination) to enhance the new generation of variables with a higher fitness value and avoid trapping in local minima [50].

#### Stepwise multiple linear regression

Stepwise regression is based on systematically adding new variables to the model. In each step a variable, which has the largest correlation with the properties vector, adds to the model or removes from it to decrease its standard deviation. Based on improvement of the regression, a partial F test judges in favour of retaining or removing this new candidate variable [50].

#### Successive projections algorithm (SPA)

The main goal of SPA is the selection of variables with the lowest collinearity. It starts with a candidate variable in the search space and calculates its orthogonal sub-space. Its strategy for selecting the next candidate variable is based on selecting the variable that has the maximum projection value on the sub-space of the previous selected variable(s). The procedure is repeated for all of the variables, and for each variable a set of N desired numbers of variables are selected. The final step is construction of forward selection MLR models. The best model is the MLR model with lowest RMSEP value [51].

#### Enhancement replacement method

The replacement method (RM) is an evolved form of the stepwise algorithm. The first time it was formulated by Duchowicz et al for the QSPR study on normal boiling points of some organic molecules [52]. It searches the pool of D (N×D) descriptors, according to the MLR procedure systematically, to find d optimal descriptors that minimize standard deviation (S):

Eq. 1 $$\text{S}=\frac{1}{\left(\text{N}-\text{d}-1\right)}\sum_{\text{i}=1}^{\text{N}}{\text{res}}_{\text{i}}^{2}$$

where N is the number of molecules in the training set and res_i_ is the difference between the experimental and the predicted properties. The RM first chooses a vector of d descriptors at random and does a linear regression [52, 53]. Then among these descriptors, each time a descriptor with the greatest standard deviation in its coefficient is substituted with all of the remaining D-d descriptors, one by one (without considering the one(s) changed previously). This procedure is repeated until the standard deviation value does not decrease by more replacements. Then the final optimal sets of d descriptors that have the smallest value of S (in equation 1) are kept. In the modified replacement method (MRM), the descriptor with the largest error is substituted even if that replacement is not accompanied by a smaller value of S. The sequence of RM-MRM-RM is called ERM. It judiciously filters the noisy variables from informative ones in a semi-full search manner [54–56].

## Figures and Tables

**Tab. 1:** Statistical parameters for comparing of different models constructed by CoMFA fields

Parameter	Traditional CoMFA	Region Focusing CoMFA	SPA-MLR	GA-MLR	SW-MLR	ERM-MLR
D^a^	6	6	13	21	15	16
r^2^_Training set_	0.9780	0.9740	0.8610	0.8620	0.9059	0.9560
r^2^_LOO-CV_	0.6040	0.6530	0.6770	0.3660	0.6071	0.8810
r^2^_LMO-CV (10group)_	0.5470	0.5920	0.6612	0.3630	0.5670	0.8700
r^2^_Test set_	0.4431	0.4470	0.3740	0.1177	0.7527	0.8630
r^2^_Evaluation set_	0.3471	0.4420	0.4590	0.4020	0.6547	0.8460
RMSEP	0.5378	0.5524	0.5715	0.7351	0.3324	0.2258
(r_0_^2^−r^2^)/r^2^	−1.241	−0.002	−0.267	−1.086	−0.008	0.000
(r^2^−r′_0_^2^)/r^2^	0.536	1.313	0.585	9.803	0.215	0.028
|r_0_^2^−r′_0_^2^′	0.67	0.59	0.12	1.02	0.16	0.02
k	1.04	0.96	0.96	0.94	1.08	0.99
k′	0.96	1.04	0.16	1.06	0.70	1.01
Predictive	No	No	No	No	Yes	Yes

a No. of latent variables or fields.

**Tab. 2:** Statistical parameters for comparing of different models constructed by CoMSIA fields

Parameter	Traditional CoMSIA	SPA-MLR	GA-MLR	SW-MLR	ERM-MLR
D^a^	6	9	14	14	17
r^2^_Training set_	0.9360	0.6930	0.5820	0.8789	0.9800
r^2^_LOO-CV_	0.3440	0.2737	0.2850	0.5900	0.8970
r^2^_LMO-CV (10group)_	0.2920	0.2832	0.2850	0.6341	0.8930
r^2^_Test set_	0.5350	0.2043	0.1360	0.7218	0.8521
r^2^_Evaluation set_	0.3920	0.5834	0.0540	0.6884	0.9080
RMSEP	0.5587	0.5814	0.6682	0.4035	0.2276
(r_0_^2^−r^2^)/r^2^	0.004	0.078	1.593	0.004	0.002
(r^2^−r′_0_^2^)/r^2^	0.579	8.010	5.000	0.089	0.014
|r_0_^2^−r′_0_^2^′	0.31	1.62	0.46	0.06	0.01
k	0.95	0.96	0.96	0.97	0.99
k′	1.05	−1.43	1.04	1.03	0.84
Predictive	No	No	No	Yes	Yes

a No. of latent variables or fields.

**Tab. 3:** Structure of 74 human HH3R antagonists

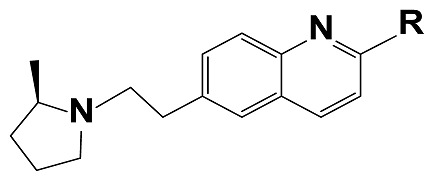		

Cpd.	Structure	pIC_50_
14	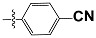	9.16
15	H	8.13
16	Me	7.92
17	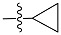	8.13
18	t-Bu	7.96
19	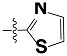	9.36
20	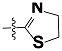	8.42
21	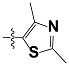	9.72
22	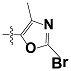	9.25
23	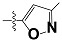	8.74
24	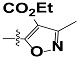	9.15
25	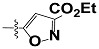	8.46
26	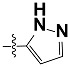	8.49
27	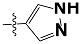	9.05
28	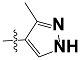	9.69
29	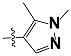	9.80
30	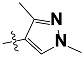	9.35
31	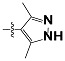	9.03
32	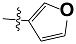	8.29
33	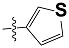	8.60
34	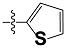	9.00
35	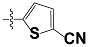	9.49
36	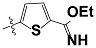	9.19
37	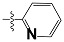	9.12
38	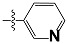	9.99
39	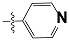	9.66
40	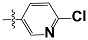	9.60
41	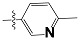	9.82
42	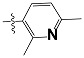	9.44
43	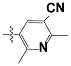	9.55
44	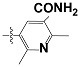	9.01
45	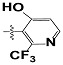	6.64
46	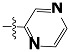	9.96
47	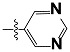	9.72
48	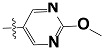	9.63
49	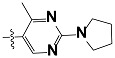	9.53
50	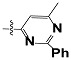	8.77
51	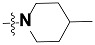	10.30
52	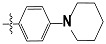	9.00
53	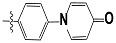	9.92
54	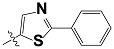	8.96
55	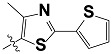	9.02
56	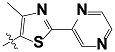	9.46
57	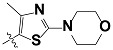	9.25
58	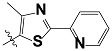	9.47
59	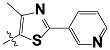	9.46
60	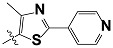	9.49
61	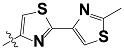	8.77
62	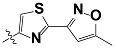	9.05
63	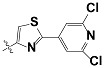	7.55
64	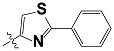	8.17
65	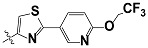	7.60
66	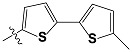	7.68
67	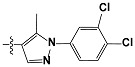	9.13
68	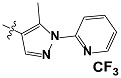	9.59
69	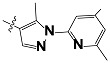	8.96
70	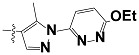	9.72
71	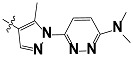	9.72
72	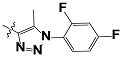	9.54
73	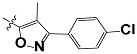	8.10
74	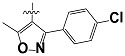	9.80
75	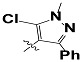	9.39
76	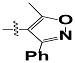	10.00
77	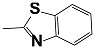	8.54
78	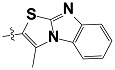	9.44
79	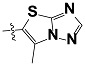	10.00
80	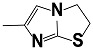	9.07
81	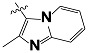	9.92
82	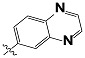	9.43
83	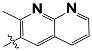	9.49
84	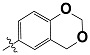	9.40
85	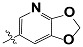	9.08
86	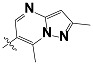	9.62
87	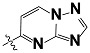	9.16
